# Reconstruction of the Second Metatarsal with Non-vascularised Fibular Graft following En-bloc Resection for Giant Cell Tumour: A Case Report

**DOI:** 10.5704/MOJ.1311.001

**Published:** 2013-11

**Authors:** P Rengsen, KL Tiong, YM Teo, TC Goh, N Sivapathasundram

**Affiliations:** Department of Orthopaedics, Hospital Melaka, Malacca, Malaysia; Department of Orthopaedics, Hospital Melaka, Malacca, Malaysia; Department of Orthopaedics, Hospital Melaka, Malacca, Malaysia; Department of Orthopaedics, Hospital Melaka, Malacca, Malaysia; Department of Orthopaedics, Hospital Melaka, Malacca, Malaysia

## Abstract

Giant cell tumour in the metatarsal of a skeletally immature person is uncommon. Adequate surgical resection in this region can be difficult to achieve as there is little space between the rays of the foot. Furthermore, there is the challenge of restoring the metatarsophalangeal articulation after resection of the tumor. We describe a technique using non-vascularised fibular graft for reconstructing the 2nd metatarsal after en-bloc resection for giant cell tumour in a 14 year old adolescent female.

**Key Words:**

Metatarsal, Giant cell tumour, Non-vascularised fibular graft, En-bloc resection

## Introduction

Giant cell tumour (GCT) remains an enigmatic bone tumour despite being extensively studied. The World Health Organization has classified GCT as “an aggressive, potentially malignant lesion”. The histology does not predict the clinical outcome, and different anatomical sites present with different management problems and difficulties^1^. Histological grading by Jaffe et. al^2^ and radiological grading by Campanacci^3^ have been shown to be unreliable in predicting the risk of local recurrence and prognosis.

GCT is estimated to represent 15% of benign and 3% to 8% of all bone tumours^1^. It is more common in China where it constitutes almost 20% of bone tumours^1^. The tumour most often appears in the second to fourth decade of life. In a review of patients in their institution in Malaysia, Ng ES et. al.^4^ reported female preponderance (59% vs 41%) with a mean age of 30.2 years. They also found that ethnic Chinese made up half (52%) of their patients. It is rare to find this tumour in the metatarsal and skeletally immature patients. Metatarsal involvement presents with the problem of achieving a wide margin for excision of the tumour as there is little space between the rays. Furthermore, the Enneking staging system classifies the hindfoot and midfoot as one compartment. As such radical resection is impossible to achieve in this scenario. There are few publications regarding the management of GCT in the metatarsal especially when preservation of the ray is attempted.

## Case Report

**Fig. 1:**
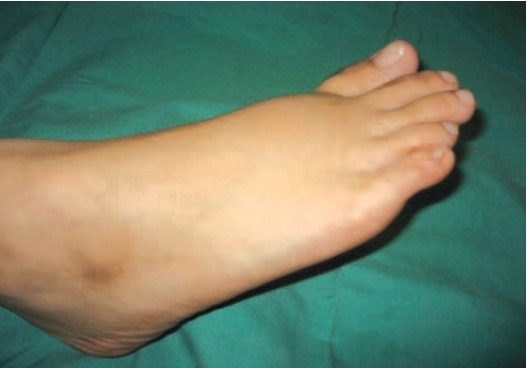
: Swelling over dorsum of right foot.

A 14 year old Chinese girl presented with swelling over the right foot (Figure 1), first noticed 10 months earlier and was increasing in size. She also had pain on walking. Examination showed a well defined swelling over the 2nd metatarsal with smooth surface, firm to hard consistency with no tethering of the overlying skin and not mobile.

**Fig. 2:**
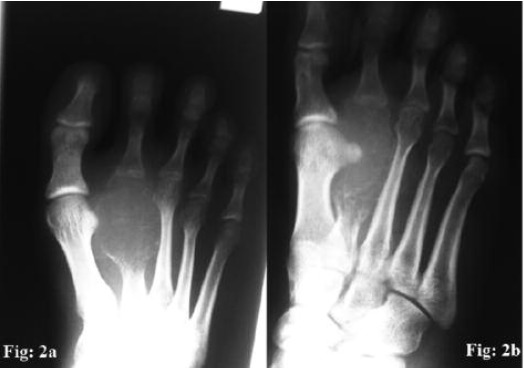
: Radiograph of right foot showing extent of tumor on (2a : AP view); (2b : Lateral view).

Radiograph of the right foot (Figure 2) revealed an expansile, osteolytic lesion of the distal two thirds of the 2nd metatarsal with soap bubble appearance. The tumour margin was ill defined and the cortices were very thinned out. Magnetic resonance imaging was suggestive of giant cell tumour of the 2nd metatarsal with surrounding oedema. CT thorax ruled out lung metastasis.

The tumour was excised en-bloc and - a marginal surgical excision as defined by Enneking was achieved. The 2nd metatarsal was reconstructed with a non-vascularised fibular graft and fixed with a small dynamic compression plate, the base of the 2nd metatarsal bone was preserved without violating the 2nd tarso-mtatarsal joint (TMT) joint. The distal part of the fibular graft was shaped to resemble the distal metatarsal condyle to fit the articulation with the proximal phalanx of the 2nd toe. The resected length was measured and restored to ensure correct soft tissue tension in the metatarsophalangeal joint. Intra-operatively the joint was stable.

**Fig. 3:**
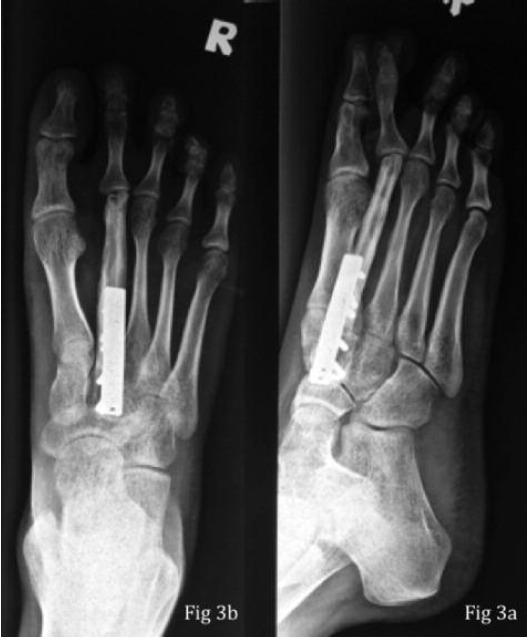
: Radiograph taken at 24 months post surgery showing incorporation of graft with no evidence of subluxation or dislocation of the second metatarso-phalangeal joint - (3a : AP view); (3b : Lateral view).

The postoperative period was uneventful and the patient was discharged on the 10th postoperative day. A cast was applied for 3 months to protect the graft and fixation. She was allowed partial weight bearing after 3 months and full weight bearing after 6 months. At the last review at 24 months the graft had incorporated and united (Figure 3). The metatarsophalangeal joint was not subluxed. Clinically there was no evidence of local recurrence. She was now pain-free and there was no tenderness over the graft or the 2nd metatarsophalangeal joint with nearly full range of motion (metatarsophalangeal joint flexion and extension of 30 degrees). There was no evidence of peroneal nerve injury and AOFAS scoring during the latest review for midfoot was 97 points and MTP/IP was 92 points.

Histopathological examination confirmed the diagnosis of high grade GCT of bone with complete resection of tumour.

## Discussion

Treatment of choice in most GCT involves curettage and bone grafting with or without the use of treatment adjuvants like post-curettage application of liquid nitrogen, methylmethacrylate, phenol, hydrogen peroxide and alcohol. Preservation of limb and motion in the affected joint is a challenge as GCT is commonly found in the epiphysis and often invades the subchondral bone and the adjacent joint.

This patient presented with a Campanacci Grade 3 tumour. In grade 3 tumours the cortex is often breached or the outer cortical shell too thinned out to make curettage possible. Enbloc resection is the treatment of choice.

There is very little written about the reconstruction of the metatarsal after excision for GCT. The first and fifth metatarsals are vital to maintaining the medial and lateral longitudinal arches of the foot and it is therefore important to reconstruct these structures when limb preservation is contemplated to ensure good functional results. The decision to reconstruct the other metatarsals must be carefully considered against donor site morbidity and technical difficulties. Treatment for GCT of the metatarsals may vary from resection alone^5^ to allograft replacement from fibula or iliac crest. Fixation methods may differ from K-wires to plates.

We describe a method which preserves metatarsophalangeal joint motion. The fibular graft can be easily shaped to resemble a metatarsal condyle with a burr. It is vital to restore the resected length to ensure joint stability. The graft is fixed with a dynamic compression plate.

## Conclusion

Although this is a single report of surgical reconstruction with non-vascularised fibular graft after excision of giant cell tumour of the metatarsal, the early outcome has been quite encouraging. We feel that this technique may be a promising surgical option in the treatment of this difficult condition.
